# Fuse me IFITM can!

**DOI:** 10.1186/s12977-014-0104-x

**Published:** 2014-11-25

**Authors:** Ariberto Fassati

**Affiliations:** The Wohl Virion Centre, Division of Infection & Immunity, Cruciform Building, University College London, 90 Gower Street, London, WC1E 6BT UK

## Two independent papers show that IFITMs proteins are incorporated into HIV-1 particles and restrict infection at an early stage

Five members of the interferon inducible transmembrane (IFITM) gene family are found in humans: IFITM1, 2, 3, 5 and 10. Typically, IFITMs 1, 2 and 3 are induced by both type I and type II interferon [1,2] although they are also constitutively expressed in cells of the upper airway and visceral pleura. IFITM 5 is not interferon-inducible and is involved in bone mineralization, and IFITM10 function is unknown. IFITM1, 2 and 3 are likely to have arisen from lineage and species-specific gene duplications. Indeed they are highly similar and highly conserved genes throughout evolution, with at least one member present in jawless fish (lampreys). The rapid expansion of the IFITM gene family during mammalian evolution was accompanied by positive selection, suggesting a broad role as antivirals. Conversely, IFITM5 and 10, which are not interferon-inducible, show no sign of gene duplications and positive selection [3,4].

IFITMs, as the name suggests, are located at membranes; IFITM1 is found mainly at the plasma membrane, IFITM2 and 3 are found mainly in intracellular compartments and endosomal membranes, although stimulation with interferon can change their distribution [5,6].

IFITMs have been shown to restrict influenza A virus (IAV) infection, in vitro and in a *Ifitm3*^*-/-*^ mouse model [7-9]. IFITM3 restricts IAV infection more potently than IFITM1 and 2, acting at the early stages of the viral replicative cycle, most likely fusion [7]. Furthermore, Caucasian and Han Chinese individuals bearing the synonymous single nucleotide polymorphism (SNP) rs12252 in the IFITM3 gene were significantly more likely to suffer from severe IAV infection and be hospitalized [8]. This SNP resulted in a truncated IFITM3 protein that is found mainly at the cell membrane instead of endosomal membranes [8]. IAV fusion and release into the cytoplasm occurs in the late endosomal compartment following acidification. Therefore it is likely that IFITM3 needs to be in the right intracellular compartment to exert its anti-IAV activity.

IFITMs proteins have been reported to restrict infection of different viruses, including West Nile and Dengue, Vescicular stomatitis, Rabies, SARS coronavirus, Semliki forest and several Bunyaviridae, with the exception of Crimean-Congo haemorragic fever virus. IFITMs inhibit virus infection by preventing efficient virus-cell fusion and it is quite possible that several IFITMs with different intracellular location have arisen to protect against viruses using different entry pathways [6]. Interestingly, Hepatitis C virus, murine leukaemia virus and Lassa virus are not restricted by IFITMs proteins, which is quite puzzling given that these viruses have overlapping entry pathways with other viruses that are restricted [6].

Whether HIV-1 is restricted by IFITMs has remained somewhat controversial. Early reports suggested that HIV-1 was not restricted, at least in HeLa-CD4 cells [7]. However a later study showed that in CD4+ T cells, replication of HIV-1 was indeed impaired [10]. In this study, IFITM 2 and 3 reduced infection at an early stage, presumably at entry, whereas IFITM1 mainly reduced virus production [10].

Now two studies, one by Tartour et a. published in Retrovirology and another by Compton et al. published in Cell Host and Microbe, convincingly demonstrate that IFITMs do indeed restrict HIV-1 infection. Remarkably, both studies report that up-regulation of IFITMs expression in target cells is insufficient to cause a significant block of HIV-1 infection. Instead, IFITMs expression at both producer and target cells was necessary for restriction. In any case, expression of IFITMs in producer cells results in more potent restriction than its expression in target cells, which was unexpected and unprecedented.

Tartour et al. and Compton et al. report that IFITMs were incorporated into HIV-1 particles, which cause their infectivity to drop. Furthermore, Compton et al. show that stimulation with interferon type I up-regulates IFITMs in primary CD4+ T cells, which, as expected, results in reduced virus replication. In a complementary fashion, Tartour et al. show that primary macrophages behaved in a similar way and produced HIV-1 particles with lower infectivity upon upregulation of IFITMs. In agreement with previous reports, Compton et al. show that HIV-1 is not really affected by IFITMs in HeLa CD4 cells and Tartour et al. show that murine leukaemia virus is insensitive to IFITMs.

Using nice imaging, the two studies demonstrate that, upon stimulation with interferon, IFITMs co-localises with sites of virus budding at the plasma membrane and with sites of Env accumulation at intracellular sites.

Both studies agree that the presence of IFITMs at the virus membrane impairs fusion, which is consistent with studies on other viruses. In addition, Compton at al. found that cell-to-cell virus transmission is also impaired by IFITMs and that this reduction is due to less efficient fusion at the virological synapse.

Several questions arise from these studies: why must IFITMs be incorporated into HIV-1 particles to be restrictive? Consensus has it that amphotropic MLV and HIV-1 fuse at the cell membrane in a pH-independent way, so why is HIV-1 susceptible and amphotropic MLV not susceptible to IFITMs? How is HIV-1 fusion inhibited? Furthermore, it will be interesting to examine the effect of the IFITM3 SNP rs12252 on HIV-1 infection: would aberrant localization of IFITM3 at the plasma membrane make HIV-1 more or less susceptible to restriction?

The two studies by Crompton et al. and Tartour et al. are important because they extend our knowledge on interferon-regulated genes that impact on HIV-1 infection and also because they point to a potentially new mechanism to explain IFITMs antiviral activity. It will be important to investigate if IFITM3 SNPs found in Han Chinese adversely affects progression to AIDS and if HIV-1 evolves differently in individuals carrying this SNP.

## References

[1] Friedman RL, Manly SP, McMahon M, Kerr IM, Stark GR (1984). Transcriptional and posttranscriptional regulation of interferon-induced gene expression in human cells. Cell.

[2] Lewin AR, Reid LE, McMahon M, Stark GR, Kerr IM (1991). Molecular analysis of a human interferon-inducible gene family. Eur J Biochem / FEBS.

[3] Zhang Z, Liu J, Li M, Yang H, Zhang C (2012). Evolutionary dynamics of the interferon-induced transmembrane gene family in vertebrates. PLoS One.

[4] Hickford D, Frankenberg S, Shaw G, Renfree MB (2012). Evolution of vertebrate interferon inducible transmembrane proteins. BMC Genomics.

[5] Diamond MS, Farzan M (2013). The broad-spectrum antiviral functions of IFIT and IFITM proteins. Nat Rev Immunol.

[6] Smith S, Weston S, Kellam P, Marsh M (2014). IFITM proteins-cellular inhibitors of viral entry. Curr Opin Virol.

[7] Brass AL, Huang IC, Benita Y, John SP, Krishnan MN, Feeley EM, Ryan BJ, Weyer JL, van der Weyden L, Fikrig E, Adams DJ, Xavier RJ, Farzan M, Elledge SJ (2009). The IFITM proteins mediate cellular resistance to influenza A H1N1 virus, West Nile virus, and dengue virus. Cell.

[8] Everitt AR, Clare S, Pertel T, John SP, Wash RS, Smith SE, Chin CR, Feeley EM, Sims JS, Adams DJ, Wise HM, Kane L, Goulding D, Digard P, Anttila V, Baillie JK, Walsh TS, Hume DA, Palotie A, Xue Y, Colonna V, Tyler-Smith C, Dunning J, Gordon SB, Smyth RL, Openshaw PJ, Dougan G, Brass AL, Kellam P (2012). IFITM3 restricts the morbidity and mortality associated with influenza. Nature.

[9] Bailey CC, Huang IC, Kam C, Farzan M (2012). Ifitm3 limits the severity of acute influenza in mice. PLoS Pathog.

[10] Lu J, Pan Q, Rong L, He W, Liu SL, Liang C (2011). The IFITM proteins inhibit HIV-1 infection. J Virol.

